# Associations of Management Factors and Environmental Conditions With the Number of Liveborn Piglets in a Commercial Pig Farm: A Retrospective Field Study

**DOI:** 10.1111/rda.70258

**Published:** 2026-06-26

**Authors:** Georgia Moutsou, Theodoros Ntallaris, Georgios Tsousis, Junwei Li, Ioannis A. Tsakmakidis, Athina Basioura

**Affiliations:** ^1^ Department of Agriculture University of Western Macedonia Florina Greece; ^2^ Clinical Sciences Swedish University of Agricultural Sciences Uppsala Sweden; ^3^ Farm Animals Clinic, School of Veterinary Medicine Aristotle University of Thessaloniki Thessaloniki Greece; ^4^ College of Veterinary Medicine, Yangzhou University Yangzhou China

**Keywords:** artificial insemination, boar semen, liveborn piglets, oestrus return, parity, pig, seasonality

## Abstract

The achievement of reproductive goals depends, among others, on semen quality, adequate control of environmental factors (e.g., heat stress) and the practice of annual sow replacement. This retrospective study examined the association of semen type (produced on the farm or purchased from artificial insemination centres), previous oestrus return, parity and thermal environment with the number of liveborn piglets. Data from 522 sows (*n* = 1035) of an industrial Greek pig farm were analysed. Except for parity, no statistical differences were observed in the number of liveborn piglets across the various parameters examined. The number of liveborn piglets was higher for second‐ to fifth‐parity sows compared to both younger and older ones. In conclusion, under the commercial conditions evaluated in the present study, the findings are consistent with previous reports and provide additional field‐based evidence supporting current herd‐management practices in commercial pig production.

## 1 Introduction

The conventional intracervical artificial insemination (AI) with liquid semen is widely used in modern pig farms, greatly contributing to the improvement of their reproductive performance (Knox 2016). Reproductive indicators such as the litter size, the number of liveborn, stillborn, mummified and weaned piglets, and the total farrowing rate per year largely determine the economic efficiency of pig farms. The number of liveborn piglets, however, represents one of the most economically important reproductive parameters in commercial pig production. It directly correlates with the potential number of weaned piglets and ultimately the final output of the pig market. Thus, optimising this parameter is a direct way to sustain the financial competitiveness of pig farming. The achievement of reproductive goals depends on proper reproductive management, such as ensuring good semen quality (Tsakmakidis et al. 2010), implementing oestrus synchronisation protocols and reducing return‐to‐oestrus rates (Shi et al. 2025).

In the last decade, a new trend has been observed, and semen doses are produced and distributed by AI centres rather than being produced on the farm. This new approach has many advantages. It minimises the costs associated with rearing boars and ensures consistently high‐quality semen doses. Boars at AI centres are housed in suitable conditions, particularly during the summer, to ensure that the temperature does not exceed 25°C and to minimise the detrimental effect of heat stress on spermatogenesis. Strict biosecurity measures are implemented to prevent the transmission of infectious diseases through semen doses and ensure the production of a high‐quality product that more farmers trust (Schulze et al. 2019). Another critical point is the assurance of the quality control of the AI semen doses, which guarantees fertilising capacity and longevity of spermatozoa by supporting their quality and functional characteristics.

Control of environmental factors, such as seasonality, humidity and ammonia levels, is of paramount importance (Einarsson et al. 2008); however, season is not easily controlled. Because temperature and humidity interact to determine the degree of heat stress experienced by pigs, the temperature–humidity index (THI) has been used as an integrated indicator of environmental heat load. Compared with a simple seasonal classification, THI provides a more precise assessment of climatic conditions and their potential impact on reproductive performance. The season of insemination may compromise the fertility of sows/gilts and boars because of heat stress (Adur et al. 2022). Besides, according to the literature, parity influences fertility. Gilts are characterised by lower reproductive performance than sows of third or higher parity (Hagan and Etim 2019). Studies have shown that the number of liveborn piglets is influenced by parity number, with the lowest number observed for primiparous sows (Knecht et al. 2015). This number increases until it reaches parity, four, six or seven, depending on breed, while litter size tends to decrease thereafter (Hagan and Etim 2019; Klimas et al. 2020).

Conditions on modern pig farms are controlled, and achieving reproductive targets is almost guaranteed. Although the impact of individual parameters has been well documented, there has been a recent industry‐wide shift towards sourcing semen from AI centres, coupled with escalating environmental challenges in Mediterranean climates. This makes it critically necessary to retest well‐known field reproductive strategies. Thus, this retrospective study aimed to evaluate whether the number of liveborn piglets remains associated with modern semen supply models, established reproductive factors (such as parity and return‐to‐oestrus) and thermal environment. This research retests management practices and provides field‐derived, actionable evidence for contemporary pig production under commercial Greek field conditions.

## 2 Materials and Methods

Ethical approval was not required since no animals were included in the study. The official data of 522 female pigs (270 gilts and 252 sows) from an industrial pig farm in Northern Greece (600 sows capacity; period 2022–2023) were processed and the following parameters were analysed for each farrowing (*n* = 1035; Table 1): (1) insemination with semen doses produced by boars on‐farm or in AI centres; (2) return to oestrus after the previous insemination; (3) parity number (Group 1: gilts/primiparous sows; Group 2: sows from the second to the fifth parity, which corresponds to animals of peak reproductive performance; Group 3: sows from the sixth parity onwards, which are older sows approaching culling age; Klimas et al. 2020); (4) thermal environment of AI (hot: THI > 70; cold: THI < 70); and (5) number of liveborn piglets. During the study period, the herd maintained an average pregnancy rate of 86.00% ± 5.48% and a farrowing rate of 83.00% ± 2.13%.

**TABLE 1:** Descriptive characteristics of artificial inseminations and sow/gilts included in the study.

Parameter	Category	Number
Type of semen	Farm‐produced semen doses	685
	Commercially available semen doses	350
Return to oestrus after previous insemination	No return	887
	Return to oestrus	148
Parity classification	Group 1: gilts and primiparous sows	270
	Group 2: sows of second to fifth parity	555
	Group 3: sows of ≥ sixth parity	210
Thermal environment of artificial insemination	Hot (THI > 70)	284
	Cold (THI < 70)	751

*Note:* This table summarises the distribution of artificial inseminations according to semen type, return to oestrus following the previous insemination, sow parity and thermal environment of artificial insemination.

All semen samples produced on the farm were collected by the gloved‐hand method and fulfilled minimum quality standards for AI semen doses. Each ejaculate was evaluated for volume (> 200 mL), sperm concentration (at least 250 × 10^6^ sperm/mL; assessed by a photometer‐SDM1, Minitube, Germany), sperm motility (> 70% mass motility; total sperm motility > 75%) and morphology (> 80% normal morphology) based on routine analysis in the farm laboratory. Sperm motility and morphology were subjectively evaluated with phase‐contrast microscopy in the farm's laboratory. All commercially available semen doses fulfilled the quality criteria, as assessed by the AI centre, and were randomly subjected to sperm motility evaluation in the farm's laboratory to confirm the adequacy of their transport and preservation conditions.

Meteorological data (monthly ambient temperature and relative humidity) for the study area during 2022–2023 were obtained from the NASA POWER database. To better characterise environmental conditions, the THI was calculated according to the equation THI = 0.8 × *T* + [(RH/100) × (*T* − 14.4)] + 46.4 (Singh et al. 2022), where *T* is monthly ambient temperature (°C) and RH is monthly relative humidity (%). Monthly THI values were calculated for the entire study period and used to characterise the thermal environment as hot when THI exceeded 70 and cold when it was below this threshold.

The management conditions, including nutrition, genetics, housing conditions, insemination protocol, veterinary supervision and environmental management, were unchanged during the study period.

Statistical analysis was performed using SASOnDemand for Academics (SAS Institute, Cary, NC, USA). Least squares means were analysed using a general linear mixed model (PROC MIXED). Model assumptions were evaluated by contacting visual and statistical diagnostics of the conditional residuals. Homogeneity of variance and linearity were assessed using a residual‐versus‐fitted value plot. The plot confirmed homoscedasticity, as it displayed a consistent vertical spread of residuals across the range of predicted values. Residual normality was evaluated using a quantile−quantile (Q−Q) plot and a histogram via the UNIVARIATE procedure. The Kolmogorov–Smirnov test indicated a significant deviation from normality (*p* = 0.01); however, visual inspection of the Q−Q plot confirmed that the middle 85% of the data points adhered strictly to the diagonal reference line, with only a minor deviation in the lower tail (negative skewness). Given the large sample size (*N* = 1035) and the documented robustness of the MIXED procedure to minor tail deviations, the residual distribution is considered acceptable, and no data transformations were undertaken. The model included semen type (farm‐collected or commercially available), return‐to‐oestrus after weaning (yes or no), parity (1, 2–5 or > 6) and thermal environment (hot or cold) as fixed effects, along with the interactions between parity and all other factors. Sow was included as a random effect. Least‐squares means were obtained for each factor category and compared using the least significant difference (LSD) test with the Tukey–Kramer adjustment for multiple comparisons. Data are presented as mean ± standard error of the mean (SEM). Differences were considered statistically significant at *p* < 0.05.

## 3 Results

**FIGURE 1:**
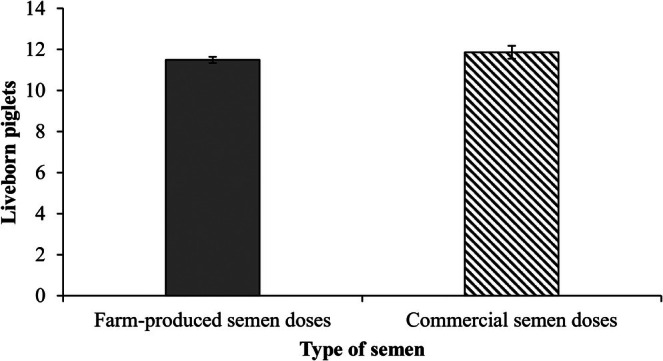
Number of liveborn piglets according to the source of artificial insemination semen doses (farm‐produced or purchased from an artificial insemination centre). Values are expressed as mean ± SEM (*n* = 1035).

**FIGURE 2:**
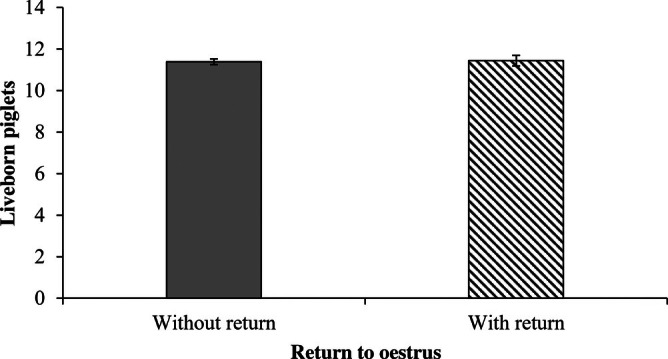
Number of liveborn piglets according to return‐to‐oestrus status. Values are expressed as mean ± SEM (*n* = 1035).

**FIGURE 3:**
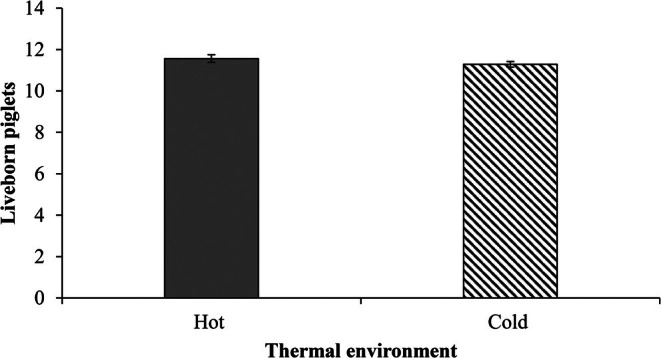
Number of liveborn piglets according to the thermal environment of insemination. Hot: THI > 70; Cold: THI < 70. Values are expressed as mean ± SEM (*n* = 1035).

**FIGURE 4:**
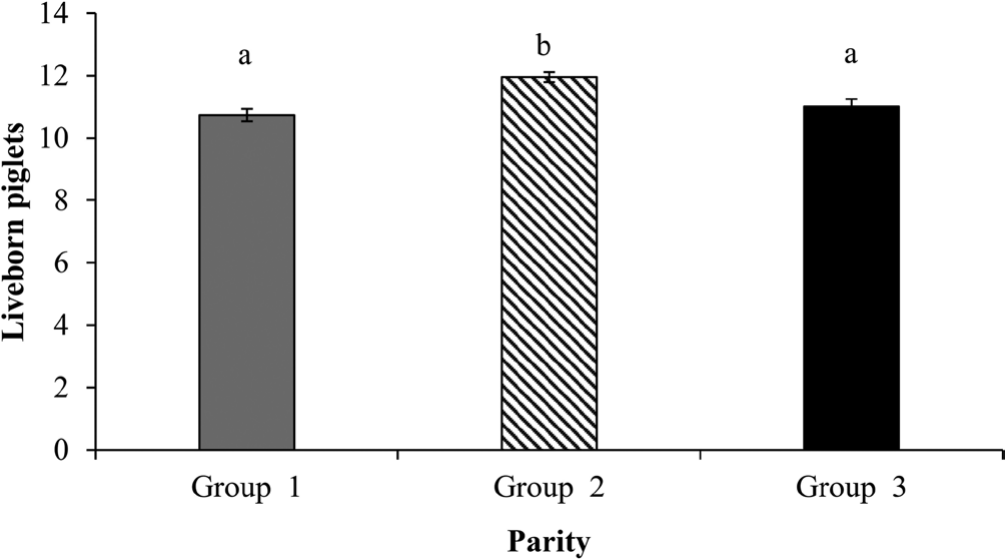
Number of liveborn piglets according to parity group. Group 1: Gilts and primiparous sows; Group 2: Sows from the second to the fifth parity; Group 3: Sows from the sixth parity onwards. Values are expressed as mean ± SEM (*n* = 1035). Different superscripts (a, b) indicate significant differences between groups (*p* < 0.05).

**FIGURE 5:**
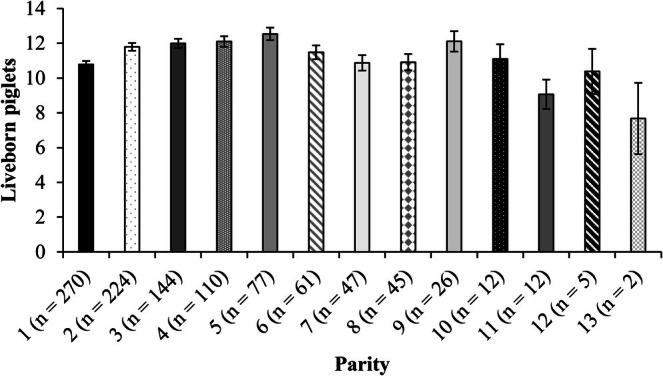
Mean number of liveborn piglets across parity groups during the study period (2022–2023). Values are expressed as mean ± SEM (*n* = 1035).

**FIGURE 6:**
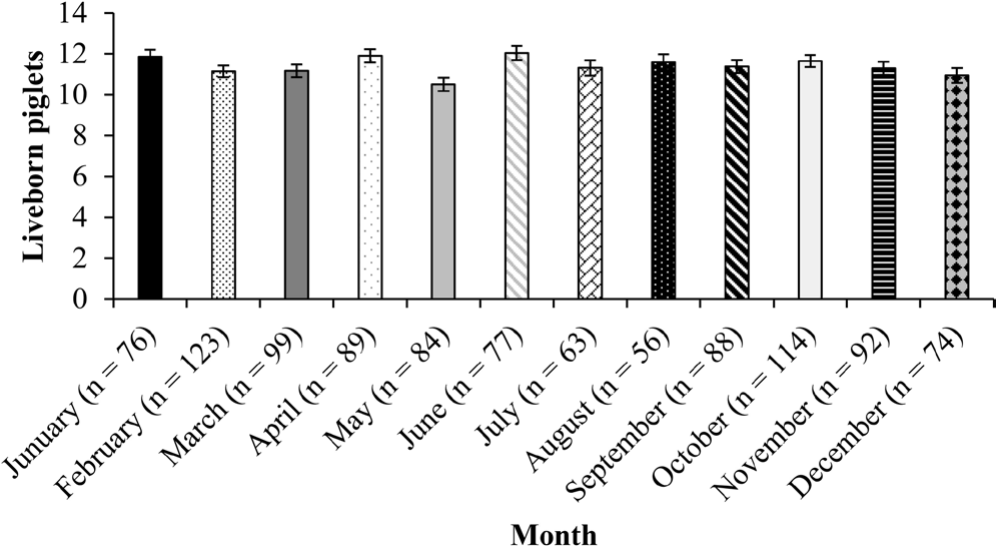
Monthly variation in the number of liveborn piglets during the study period (years 2022–2023). Values are expressed as mean ± SEM (*n* = 1035).

Figure 1 illustrates the mean number of liveborn piglets obtained following insemination with semen produced on‐farm and semen obtained from a commercial AI centre. No statistically significant difference was observed between semen sources (*p* > 0.05), indicating that both semen supply systems achieved comparable reproductive outcomes under the management conditions of the studied farm. Figure 2 presents the number of liveborn piglets according to the occurrence of return to oestrus following AI, and no statistical difference was observed between the two categories. Figure 3 presents the number of liveborn piglets according to the thermal environment of insemination. Although numerically different values were observed between THI categories, these differences were not statistically significant (*p* > 0.05). As shown in Figure 4, parity is significantly associated with the number of liveborn piglets (*p* < 0.05). Sows in parity 2–5 (Group 2) produced the largest litters compared to gilts/primiparous sows (Group 1; *p* = 0.001) and sows of parity ≥ 6 (Group 3; *p* = 0.002). No statistically significant interaction was found between the parity and the other factors (semen‐supply system, return to oestrus after insemination and thermal environment). Figure 5 shows the number of liveborn piglets per parity, and Figure 6 illustrates the monthly variation in the number of liveborn piglets throughout 2022 and 2023. Although fluctuations were observed between months, no consistent temporal trend indicating a progressive increase or decrease in the number of live births was evident during the study period.

Monthly THI values ranged from 41.5 to 76.4 during the study period (Table 2). The highest values were recorded during July and August of both years, whereas the lowest values occurred during the winter months. THI values above 70 were observed throughout most summer months, indicating periods of potential heat stress.

**TABLE 2:** Monthly ambient temperature, relative humidity and temperature–humidity index (THI) values for the study area during the experimental period (2022–2023).

Month	Year	Temperature (°C)	Relative humidity (%)	THI
January	2022	4.08	78.95	41.5
	2023	7.28	79.74	46.5
February	2022	6.72	78.24	45.8
	2023	6.47	68.98	46.1
March	2022	6.13	69.27	45.6
	2023	10.24	71.19	51.6
April	2022	13.32	69.79	56.3
	2023	12.37	69.11	54.9
May	2022	20.46	56.69	66.2
	2023	17.44	68.97	62.4
June	2022	25.79	54.33	73.2
	2023	22.85	63.84	70.1
July	2022	27.64	43.36	74.3
	2023	29.12	45.66	76.4
August	2022	27.39	47.84	74.5
	2023	28.08	44.03	74.9
September	2022	23.60	50.00	70.0
	2023	23.31	57.86	70.2
October	2022	17.52	56.12	62.2
	2023	19.54	60.64	65.1
November	2022	13.35	73.58	56.3
	2023	13.40	70.26	56.4
December	2022	9.48	82.45	49.9
	2023	8.32	78.71	48.3

*Note:* Climatic data were obtained from the NASA POWER database. THI was calculated to provide a quantitative assessment of environmental heat load. THI was calculated according to the equation THI = 0.8 × *T* + [(RH/100) × (*T* − 14.4)] + 46.4, where *T* is monthly ambient temperature (°C) and RH is monthly relative humidity (%).

## 4 Discussion

This retrospective study was conducted in collaboration with a commercial pig farm to evaluate, under field conditions, the associations of semen source, return to oestrus status, seasonality and parity with the number of liveborn piglets. During the study period, the overall pregnancy and farrowing rates were 86.00% ± 5.48% and 83.00% ± 2.13%, respectively. These indicators were monitored routinely on the farm and remained relatively stable throughout the study period. These values are within the range typically reported for well‐managed commercial pig herds and indicate satisfactory reproductive efficiency during the study period. Furthermore, the percentages of stillborn piglets and pre‐weaning mortality remained consistently low (< 2% and < 1%, respectively). Because these parameters showed limited variability throughout the study period, they were not considered suitable for meaningful statistical evaluation within the scope of the present study. Consequently, the number of liveborn piglets was selected as the primary reproductive outcome for analysis. The number of liveborn piglets constitutes the primary driver of profitability and sustainability in modern commercial pig farming. While other reproductive factors, such as farrowing rates, indicate the success of fertilisation processing, the number of liveborn piglets quantifies the actual productive yield.

In recent years, the market has shifted towards supplying AI semen doses from AI centres, rather than producing them on the farm. In the present study, the number of liveborn piglets was not different between sows inseminated with commercially available semen doses or semen doses produced on the farm. The absence of differences between semen sources is consistent with adequate semen quality and handling procedures for both farm‐produced and commercially supplied semen doses. These findings indicate that semen sources were associated with similar numbers of liveborn piglets under the management conditions of the studied farm.

Return‐to‐oestrus is an important reproductive indicator, and an elevated return rate is associated with an increase in non‐productive days and suboptimal reproductive outcomes in sows (Tani et al. 2016). Seasonal variation in return rates has been reported, with peak return rates occurring during the summer period (Nguyen et al. 2026). Differences in return rates are also associated with parity, as primiparous sows exhibit higher return rates than multiparous sows (Tani et al. 2016). However, in the present study, no differences were observed in the number of liveborn piglets between females inseminated after returning to oestrus and those conceiving after the first insemination. Furthermore, no interaction was detected between return‐to‐oestrus and either parity or THI categories, indicating that the relationship between return status was consistent across parity groups and thermal environment. This finding suggests that, under the management and environmental conditions of the studied farm, successful conception following a return‐to‐oestrus was not associated with a reduction in the number of liveborn piglets, regardless of the sow's age or the thermal conditions of insemination. The lower number of liveborn piglets observed in older sows is consistent with the replacement strategies commonly applied in commercial pig production systems.

In the present field study, the number of liveborn piglets was found to be higher between the second and fifth parity than in the first or subsequent ones. The parity‐related pattern observed is consistent with previous reports indicating low reproductive performance in primiparous sows, peak reproductive performance during middle parities and a subsequent decline in older sows (Hagan and Etim 2019; Klimas et al. 2020). This finding is consistent with current commercial herd‐management practices regarding sow replacement. The absence of interaction between parity and semen source suggests that the association between semen source and the number of liveborn piglets was consistent across parity groups.

THI was used as an integrated indicator of environmental heat load that is widely employed to describe the combined effects of temperature and humidity and therefore provides a more detailed characterisation of environmental conditions (Singh et al. 2022). However, the calculated THI values demonstrated that environmental conditions varied considerably within months. Monthly THI ranged from 41.5 to 76.4, with values exceeding 70 during most summer months. The mean THI during the defined hot conditions (June–September) was approximately 72.5, whereas it was approximately 52.3 during the cold (October–May). Therefore, THI data suggest that animals experienced varying levels of thermal challenge during the study period. No differences in the number of liveborn piglets, however, were observed between the two THI categories, which may indicate that environmental and management conditions operating on the farm contributed to mitigating the potential adverse effects of heat stress.

In conclusion, under the conditions of this retrospective observational study, the number of liveborn piglets obtained with farm‐produced semen doses was comparable to that obtained with commercially available semen doses. This finding is consistent with effective semen production and handling procedures under the conditions of the studied farm. The inclusion of THI data further indicated that the number of liveborn piglets remained relatively stable despite periods of moderate environmental heat load, suggesting that factors operating under the farm conditions may have mitigated the adverse effects of heat stress. Nevertheless, elevated temperatures during the summer period may negatively affect semen quality and fertility if appropriate environmental control measures are not implemented. Under such circumstances, the use of semen doses obtained from AI centres may represent a practical option for maintaining acceptable reproductive outcomes. Under the field conditions evaluated, the number of liveborn piglets was lower in sows of sixth parity and above, supporting current herd‐management practices. These findings are consistent with previously reported reproductive patterns and provide additional field‐based evidence from a contemporary commercial pig production system. However, the results should be interpreted with caution, as the study was conducted on a single commercial farm and the observational, retrospective design does not permit causal inference. Future studies involving multiple commercial farms and a broader range of reproductive and environmental parameters are warranted to validate these findings and improve their generalisability.

## Author Contributions

**Georgia Moutsou:** data curation, investigation, visualisation, writing – original draft. **Theodoros Ntallaris:** methodology, writing – review and editing. **Georgios Tsousis:** formal analysis, visualisation, validation, writing – original draft, writing – review and editing. **Junwei Li:** methodology, writing – review and editing. **Ioannis A. Tsakmakidis:** methodology, investigation, visualisation, validation, writing – review and editing. **Athina Basioura:** conceptualisation, supervision, methodology, investigation, visualisation, validation, writing – original draft, writing – review and editing.

## Funding

The authors have nothing to report.

## Ethics Statement

The authors have nothing to report.

## Conflicts of Interest

The authors declare no conflicts of interest.

## Data Availability

The authors have nothing to report.
